# Molecular Mechanisms of Cadherin Function During Cortical Migration

**DOI:** 10.3389/fcell.2020.588152

**Published:** 2020-09-15

**Authors:** Isabel Martinez-Garay

**Affiliations:** Division of Neuroscience, School of Biosciences, Cardiff University, Cardiff, United Kingdom

**Keywords:** cerebral cortex, neuron, migration, cell surface, adhesion molecules, CDH2, molecular mechanism

## Abstract

During development of the cerebral cortex, different types of neurons migrate from distinct origins to create the different cortical layers and settle within them. Along their way, migrating neurons use cell adhesion molecules on their surface to interact with other cells that will play critical roles to ensure that migration is successful. Radially migrating projection neurons interact primarily with radial glia and Cajal-Retzius cells, whereas interneurons originating in the subpallium follow a longer, tangential route and encounter additional cellular substrates before reaching the cortex. Cell-cell adhesion is therefore essential for the correct migration of cortical neurons. Several members of the cadherin superfamily of cell adhesion proteins, which mediate cellular interactions through calcium-dependent, mostly homophilic binding, have been shown to play important roles during neuronal migration of both projection neurons and interneurons. Although several classical cadherins and protocadherins are involved in this process, the most prominent is CDH2. This mini review will explore the cellular and molecular mechanisms underpinning cadherin function during cortical migration, including recent advances in our understanding of the control of adhesive strength through regulation of cadherin surface levels.

## Introduction

**FIGURE 1:**
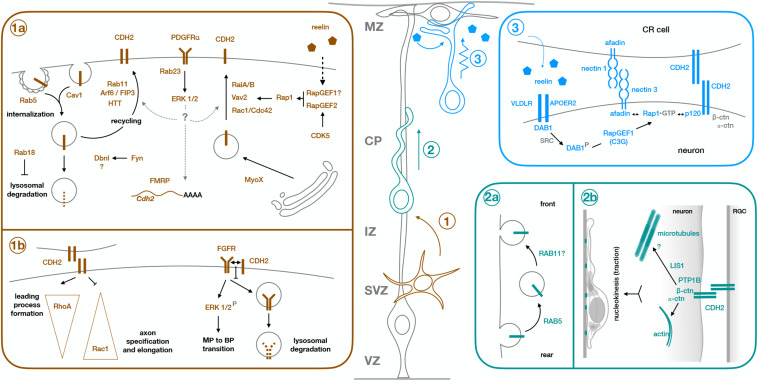
Molecular mechanisms of CDH2 during the different steps of radial neuronal migration. Projection neurons are born from Radial Glia progenitors (gray cell, RGC) in the ventricular zone (VZ) or from intermediate progenitors in the subventricular zone (SVZ). They undergo multipolar migration while in the SVZ and intermediate zone (IZ) (step 1, brown cell), before developing a leading process and an axon, and acquiring bipolar morphology. They enter the cortical plate (CP) as bipolar cells and use the processes of the Radial Glia progenitors as scaffold to migrate along (step 2, green cell). Once they are close enough to the marginal zone (MZ) for their leading process to make contact with RGC endfeet and Cajal-Retzius cells (CR cell), neurons switch to terminal translocation for the last phase of their migration (step 3, blue cell). Early born neurons do not need a locomoting step because the cortical wall is still very thin at the time of their migration, so they ascend directly through somal translocation after becoming bipolar. Box **1a** shows the molecular mechanisms regulating CDH2 total or surface expression during multipolar migration and multipolar to bipolar transition (step 1), while box **1b** shows downstream effects of CDH2 at this stage. Similarly, boxes **2a** and **2b** show regulation of CDH2 and downstream effects during glia-guided locomotion (step 2), and box **3** shows regulation of CDH2 surface levels during somal translocation (step 3). For details, see text.

Whilst cellular movements are an essential developmental feature of most organs, the orchestration of such movements in the nervous system is particularly relevant, as much of the central nervous system is organized in distinct layers that typically share functional properties. In the brain, neurons are generated in proliferative areas close to the ventricles and subsequently migrate to reach their definitive positions in different regions. Neocortical projection neurons migrate radially from the pallial ventricular or subventricular zone (Haubensak et al., 2004; Noctor et al., 2004), whereas cortical interneurons migrate tangentially from the ventral telencephalon (de Carlos et al., 1996; Anderson et al., 1997; reviewed in Marín and Rubenstein, 2001; Ayala et al., 2007; see also Figure 1). During their journey, they all navigate through complex extracellular environments that include other cells, which play critical roles in ensuring a successful migration. Interactions between Cajal-Retzius cells in the marginal zone of the cortex and migrating projection neurons are crucial for somal and terminal translocation (Gil-Sanz et al., 2013). Likewise, locomoting neurons use the basal processes of radial glia progenitors as a scaffold to migrate along (Rakic, 1972). Contact between migrating neurons and their cellular substrates is mediated by different cell-cell adhesion molecules, including the cadherin superfamily, with over 100 members expressed preferentially or exclusively in the nervous system in vertebrates. Cadherins are calcium-dependent cell-cell adhesion molecules, characterized by the presence of a variable number of extracellular cadherin repeats, that can be broadly subdivided into three main subfamilies (Sotomayor et al., 2014): the classical cadherins and the clustered and non-clustered protocadherins. Members of all three subfamilies have been shown to play a role in cortical migration. Alpha clustered protocadherins regulate radial migration through a pathway involving WAVE, Pyk2 kinase and the small GTPase Rac1 (Fan et al., 2018). Interfering with protocadherins DCHS1 and FAT4 also affects neuronal positioning (Cappello et al., 2013) and leads to migration defects in human projection neurons (Klaus et al., 2019). Protocadherin 20 determines the final position of layer 4 neurons in mice (Oishi et al., 2016), while another protocadherin, Celsr3, is required for interneuron migration (Ying et al., 2009). However, the cadherin with the best characterized role in cortical migration is the classical cadherin CDH2 (N-cadherin), which will therefore be the focus of this review.

## CDH2 Roles During Neuronal Migration

After initial bipolar migration to the subventricular zone and lower intermediate zone, newborn neurons adopt a multipolar morphology before developing leading and trailing processes and becoming bipolar in the upper intermediate zone to progress into the cortical plate (Nadarajah et al., 2001; Tabata and Nakajima, 2003; Noctor et al., 2004). The multipolar to bipolar (MBP) transition is a crucial step that involves many different proteins (Cooper, 2014). Failure to form a leading process impedes migration into the cortical plate, which proceeds first through somal translocation for early born neurons and then by glia-guided locomotion (Kawauchi, 2015) followed by terminal translocation as the cortical wall grows in thickness (Nadarajah et al., 2001; Figure 1). Interneurons first migrate tangentially from the ganglionic eminences into the cortex and subsequently switch to radial migration to invade the cortical plate (Marín and Rubenstein, 2001).

CDH2 has been involved in most of these steps. As one of the main components of adherens junctions keeping radial glia endfeet connected (Kadowaki et al., 2007), CDH2 first needs to be downregulated to allow detachment of newborn neurons from the adherens junction belt and their subsequent delamination (Rousso et al., 2012). Once neurons reach the subventricular zone/intermediate zone, CDH2 is needed for correct polarization in the radial direction and for the specification of the leading and trailing processes (Jossin and Cooper, 2011; Gärtner et al., 2012; Xu et al., 2015). Further to its role in MBP transition, CDH2-mediated adhesion between migrating neurons and radial glia fibers is needed for the locomoting phase (Shikanai et al., 2011), with adhesion sites providing traction for the forward movement of the nucleus (Martinez-Garay et al., 2016). Similarly, attachment of the leading process to radial glia endfeet and Cajal-Retzius cells in the marginal zone for somal or terminal translocation is also dependent on CDH2 (Franco et al., 2011; Gil-Sanz et al., 2013). The following sections will focus on the molecular mechanisms known to operate around CDH2 during those different roles, with a significant part of the review devoted to regulation of CDH2 surface levels, as this topic has been more extensively studied.

## Regulation of CDH2 Levels During Radial Migration

The strength of cadherin-mediated adhesions depends mainly on the levels of cadherins at the cell surface. These levels are, in turn, regulated by the balance between the rate of biosynthesis and of delivery to the plasma membrane, and turnover of cadherin molecules by endocytic processes. Recent reviews provide a comprehensive account of cadherin trafficking (Cadwell et al., 2016; West and Harris, 2016), so this section will mainly focus on the specifics of CDH2 regulation in migrating neurons (Table 1).

**TABLE 1:** Regulators of CDH2 levels in migrating neurons.

Protein	Paradigm^1^	Effect on CDH2 levels	Migration stage	References
Afadin	KD (shRNA) (*in vitro*)^2^	↓ surface	ST	Gil-Sanz et al., 2013
ARF6	KD (shRNA) iuEP	↑ intracellular	MBPT	Hara et al., 2016
Caveolin 1	KD (shRNA) (*in vitro*)	↑ surface	MBPT	Shikanai et al., 2018
CDK5	*Cdk5* ko (*in vitro*) DNCDK5 (K33T) (293T cells)	↓ levels = total levels ↑ intracellular	MBPT MBPT	Ye et al., 2014 Lee et al., 2019
DBNL	KD (shRNA) (*in vitro*) FIP3 ΔABD (*in vitro*) FIP3 ΔRBD (*in vitro*)	↓ surface	MBPT	Inoue et al., 2019
ERK 1/2	Inhibition of phosphorylation with PD98059, U0126 (*in vitro*)	↓ levels	MBPT	Hor and Goh, 2018
FIP3	KD (shRNA) iuEP	↑ intracellular	MBPT	Hara et al., 2016
FMRP	*Fmr1* ko (WB, RT-qPCR)	↓ Cdh2 mRNA ↓ CDH2 protein	MBPT	La Fata et al., 2014
HTT	*Htt^*lox/lox*^;Nex^*CRE/*^*^+^ ND:CRE-GFP iuEP into *Htt*^*lox/lox*^ (WB, *in vitro*)	= total levels ↓ surface ↑ perinuclear	MBPT	Barnat et al., 2016
MYO10	KD (shRNA) (293T cells)	↓ surface ↑ cytoplasmic	MBPT	Lai et al., 2015
Nectin 1/3	Primary neurons cultured on Nectin-1 vs. PLL	↑ surface	ST	Gil-Sanz et al., 2013
RAB5	DNRAB5 (S34N) (*in vitro*) KD (shRNA) (*in vitro*)	↑ surface	MBPT GGL	Kawauchi et al., 2010
RAB11	DNRAB11 (S25N) (*in vitro*, iuEP) KD (shRNA) (*in vitro*, iuEP) DNRAB11 (S22N) (*in vitro*, iuEP)	↑ perinuclear ↑ perinuclear	MBPT GGL MBPT	Kawauchi et al., 2010 Barnat et al., 2016
RAB18	KD (shRNA) (*in vitro*)	↓ surface	MBPT	Wu et al., 2016
RAB23	KD (shRNA) (*in vitro*, iuEP) *Rab23^*lox/lox*^;Emx1^*CRE/*^*^+^ (WB)	↓ protein (FL and cytopl. fragment) ↓ Cdh2 mRNA	MBPT	Hor and Goh, 2018
RAP1	conditional KO (Rap1a + b) (IHC of EP brain slices) RAP1GAP OE (*in vitro*, iuEP) RAPGEF2 KD (shRNA), RAPGEF2 S1124A (*in vitro*)	↓ levels ↓ surface ↓ surface	MBPT MBPT MBPT	Shah et al., 2016 Jossin and Cooper, 2011 Ye et al., 2014
REELIN	Application of reelin to *reeler* neurons cultured on Nectin-1	↑ surface	ST	Gil-Sanz et al., 2013

*^1^Note that in some of the references in the table, other experimental paradigms were used in addition to the ones listed. However, the table reflects the ones used when assessing effects on CDH2 levels, rather than migration as a whole. ^2^In vitro refers to cortical primary neurons cultured in vitro, either from mutant brains, electroporated brains, or obtained from control brains and transfected. KD, knockdown; shRNA, short hairpin RNA; iuEP, in utero electroporation; MBPT, multipolar to bipolar transition; GGL, glia-guided locomotion; ST, somal or terminal translocation; FL, full length.*

Newly synthesized CDH2 in the endoplasmic reticulum binds to β-catenin and p120-catenin (Wahl et al., 2003), which regulates subsequent transport to the Golgi and the plasma membrane through interaction with kinesin motors (Mary et al., 2002; Chen et al., 2003; Teng et al., 2005; Wehrendt et al., 2016). Internalization of surface CDH2 is mediated by clathrin-dependent and independent endocytic mechanisms (Sharma and Henderson, 2007; Tai et al., 2007; Chen and Tai, 2017) and binding of β-catenin and p120-catenin to CDH2 regulates this process, partly by masking key residues needed for endocytosis (Chen and Tai, 2017). Internalized receptors can be either recycled back to the cell membrane or routed to the lysosome for degradation in an endosomal sorting pathway mainly controlled by Rab GTPases and their effectors (Wandinger-Ness and Zerial, 2014).

An intriguing possibility to further modulate cadherin adhesion is allosteric regulation. This form of inside-out signaling, well characterized in integrin-mediated adhesion (Hynes, 2002), has so far been demonstrated for CDH1 (Shashikanth et al., 2015), but whether it could provide a way to quickly control CDH2 adhesive strength is not yet known.

### Regulation of CDH2 Total Levels

In migrating neurons, one way to adjust CDH2 levels is through regulation of *Cdh2* mRNA. FMRP binds *Cdh2* mRNA and in *Fmr1*^–/–^ animals, which display increased numbers of multipolar neurons in the intermediate zone, *Cdh2* mRNA levels are reduced and the migration defect is rescued by overexpression of CDH2 (La Fata et al., 2014).

Similarly, downregulation of the small GTPase Rab23 also leads to a decrease of CDH2 levels in newborn neurons (Hor and Goh, 2018). This reduction, which is apparent at contact surfaces between interacting neurons, affects both the full-length protein and its processed cytoplasmic fragment, and is accompanied by a decrease in mRNA levels. Phenotypically, it leads to an increase in the number of multipolar neurons in the intermediate zone that fail to progress into the cortical plate. At the molecular level, diminished Rab23 levels seem to impair activation of PDGFRα and its subsequent phosphorylation of ERK1/2; indeed, pharmacological inhibition of ERK1/2 also led to a reduction in CDH2 levels (Hor and Goh, 2018).

Another Rab GTPase involved in the control of total CDH2 levels is Rab18. Knockdown of this GTPase or interference with its function leads to a migration defect in the intermediate zone that can also be partially rescued by overexpression of CDH2. In this case, reduction of surface CDH2 levels as revealed by TIRF microscopy are correlated with a global decrease in CDH2 through lysosomal degradation rather than protein expression (Wu et al., 2016).

### Regulation of CDH2 Surface Levels

In addition to adjusting global CDH2 levels, migrating neurons also dynamically regulate CDH2 surface levels through several pathways, allowing for more flexible responses to varying extracellular environments (Solecki, 2012; Figures 1,1a,2a,3).

#### Regulation by Rab GTPases

As main regulators of endosomal trafficking, Rab GTPases are ideal candidates to control CDH2 surface levels. Kawauchi et al showed that Rab11 and Rab5 play opposing but essential roles in the trafficking of CDH2 to and from the plasma membrane (Kawauchi et al., 2010; Kawauchi, 2011; Figures 1,1a,2a). Knockdown of Rab5 reduces Rab5-mediated endocytosis, leading to an accumulation of cells in the intermediate zone with abnormal morphology and increased adhesion between neurons and radial glia processes. This migration defect is partially rescued through slight downregulation of CDH2 levels, implicating Rab5 in the internalization of CDH2 (Kawauchi et al., 2010). This same study showed that Rab11 is needed for correct recycling of CDH2 back to the cell membrane after endocytosis. Electroporation of dominant negative (DN)-Rab11 results in redistribution of CDH2 from the cell surface to perinuclear regions and its accumulation in transferrin positive vesicular compartments, concomitant with a delay in radial migration.

Two other proteins have been shown to regulate CDH2 surface levels through an interplay with Rab11. Lack of HTT, which is expressed in the upper intermediate zone and the cortical plate, results in migration defects with neurons failing to acquire a bipolar morphology and showing abnormal interaction with radial glia fibers. HTT and CDH2 normally co-localize in the leading process of migrating neurons, but in the absence of HTT, CDH2 is relocated to transferrin positive vesicles in the perinuclear region and CDH2 surface levels are significantly reduced. Interestingly, co-expression of Rab11 or its constitutively active form rescues the migration defect of HTT depleted cells, as does overexpression of CDH2 (Barnat et al., 2016; Figures 1,1a).

The small GTPase Arf6 localizes to a subpopulation of early and recycling endosomes and interfering with its function disrupts migration and increases intracellular CDH2. This increase is not due to changes in the internalization of CDH2, but rather to its defective recycling back to the cell surface. Only Arf6 capable of interacting with its effector FIP3 and with Rab11 can rescue the migration defect of Arf6 knockdown (Hara et al., 2016; Figures 1,1a).

#### Regulation by Rap1

The small GTPase Rap1 is one of the major regulators of CDH2 surface levels, both during multipolar migration and the MBP transition, and during somal and terminal translocation (Figures 1,1a,3). Jossin and Cooper showed that interfering with normal Rap1 function delays the progression of neurons to a bipolar state (Jossin and Cooper, 2011). This cell-autonomous defect is due to reduced surface levels of CDH2 and can be rescued by CDH2 overexpression. The molecular mechanism linking Rap1 to CDH2 involves Vav2, an activator for Rac1, and other small GTPases (RalA/B and Rac1/Cdc42). Interestingly, reelin signaling acts upstream of Rap1 to regulate CDH2 membrane levels in the intermediate zone, as it does in the upper cortical plate during somal or terminal translocation (Franco et al., 2011; Jossin and Cooper, 2011). In translocating neurons, reelin activates Rap1 through RapGEF1 (C3G) (Franco et al., 2011), but CDH2 recruitment to the cell surface is dependent on nectin-mediated adhesion between migrating neurons and Cajal-Retzius cells (Gil-Sanz et al., 2013). CDH2 overexpression rescues migration defects caused by downregulation of nectin 3 or its effector Afadin, which interacts with Rap1 and p120 catenin, providing a link between Rap1 activation and CDH2 (Gil-Sanz et al., 2013; Figures 1,3). Recently, the reelin-induced increase in CDH2 surface levels in translocating neurons has been shown to be transient rather than sustained, but the mechanism behind it remains unknown (Matsunaga et al., 2017).

Another molecule acting upstream of Rap1 to regulate CDH2 surface levels is CDK5. Despite initial reports of CDK5 negatively regulating cadherin-mediated adhesion and the interaction between β-catenin and CDH2 (Kwon et al., 2000), this negative effect of CDK5 on CDH2 adhesion has not been reproduced *in vivo*. CDK5 phosphorylates RapGEF2 in the developing cortex, enhancing its GEF activity toward Rap1 (Ye et al., 2014). RapGEF2 is strongest expressed in the upper intermediate zone and is required for MBP transition, since its knockdown leads to multipolar neurons that accumulate in the lower intermediate zone. Membrane CDH2 levels are reduced in the *Cdk5*^–/–^ cortex and in neurons electroporated with either shRNA against RapGEF2, or its non-phosphorylatable form S1124A, and migration defects caused by RapGEF2 inhibition can be rescued by moderate overexpression of CDH2 (Ye et al., 2014). Similar results were reported in a recent study that looked at the link between CDK5 and CDH2 in the developing cortex, which showed that migration defects caused by *in utero* electroporation of DNCDK5 at E14.5 could be partially rescued by co-electroporation with CDH2 (Lee et al., 2019; Figures 1,1a).

#### Regulation by Other Proteins

In addition to small GTPases, other proteins linked to endocytosis and the actin cytoskeleton are involved in the control of CDH2 surface levels in migrating neurons. The actin motor MYO10 interacts with CDH2 through its FERM domain and seems to mediate its transport from the Golgi to the plasma membrane. Downregulation of this unconventional myosin reduces surface CDH2, but not its total levels. This leads to accumulation of cells in the intermediate zone that display disrupted interaction with radial glia fibers and decreased locomoting speed in those neurons that make it to the cortical plate. MYO10 also colocalizes with markers for early, late and recycling endosomes, suggesting that it might play a role in the trafficking of CDH2-containing endosomes (Lai et al., 2015). Drebrin-like (DBNL) is an adaptor protein that binds F-actin and Dynamin 1 and is thus involved in receptor-mediated endocytosis and remodeling of the actin cytoskeleton. As in the case of MYO10, knockdown of DBNL reduces CDH2 levels at the cell surface. How this reduction is brought about at the molecular or cellular level is not known, but it seems to involve phosphorylation of two Tyr residues in Dbnl by Fyn. Dbnl-deficient neurons complete the MBP transition despite defects in neurite extension and polarization, but do not enter the cortical plate (Inoue et al., 2019). In both cases, overexpression of CDH2 partially rescues the migration defects (Figures 1,1a).

Despite neurons not displaying caveolae, caveolin 1 is expressed in the developing cortex, particularly in the neurites of multipolar cells in the intermediate zone, where it is involved in clathrin-independent endocytosis. Downregulation of caveolin 1 increases the ratio of surface to total levels of two adhesion proteins: CDH2 and L1CAM, while decreasing their levels in early endosomes, suggesting that caveolin 1 is needed for their internalization (Shikanai et al., 2018). Neurons deficient in caveolin 1 acquire bipolar morphology, but their leading processes are shorter and more branched than in control neurons, with increased immature neurites that are retained even after leading process formation (Figures 1,1a).

## Mechanisms Downstream of CDH2

Compared to the wealth of information about the regulation of CDH2 surface levels in migrating neurons, much less is known about the mechanisms operating downstream of CDH2. With regards to the initial specification of neuronal processes, *in vitro* experiments suggest that opposing gradients of active RhoA at the leading process and Rac1 in the axon are established as a consequence of CDH2-mediated contact (Xu et al., 2015). Although the exact mechanism by which this is accomplished remains unclear, work in C2C12 fibroblasts has shown that CDH2 engagement decreases Rac1 and Cdc42 activity and increases RhoA activity (Charrasse et al., 2002). This could explain the formation of the gradients, as sites of CDH2 adhesion between neurons and radial glia fibers provide a positional cue for the development of the leading process while directing axonal formation to the opposite pole of the cell (Gärtner et al., 2012; Xu et al., 2015; Figures 1,1b).

A second mechanism at play during CDH2-mediated neuronal polarization involves its interaction with FGFRs to prevent their ubiquitination and subsequent lysosomal degradation. This interaction happens in *cis* and, surprisingly, does not require CDH2-mediated adhesion. As a result of higher levels of FGFR at the cell membrane, the ERK1/2 signaling pathway is activated. Since similar results in ERK1/2 activation are obtained by long treatment with reelin *in vitro*, FGFR and ERK1/2 can be considered downstream components of the reelin – Rap1 – CDH2 axis (Kon et al., 2019; Figures 1,1b).

Glia-guided locomotion requires CDH2-mediated adhesion and its connection to the actin cytoskeleton through alpha-N-catenin. When CDH2 (and CDH4) adhesion is weakened by electroporation of different dominant negative forms, neurons can still polarize and extend leading processes into the cortical plate. However, nucleokinesis fails and the leading processes become twice as long as in control neurons. The collapse of the processes upon induced actomyosin contraction indicates that CDH2-mediated contacts between neurons and radial glia fibers probably act as sites for traction generation to allow nuclear movement. In addition, neurons electroporated with DNCDH also show an abnormal accumulation of LIS1 in the leading process, hinting to a potential mechanism involving microtubules downstream of CDH2 in migrating neurons (Martinez-Garay et al., 2016; Figures 1,2b).

## Role of CDH2 in Interneuron Migration

CDH2 is also involved in the generation and migration of cortical interneurons. As well as maintaining the organization of the neuroepithelium through adherens junctions, CDH2 engagement stimulates interneuron motility (Luccardini et al., 2013). Interneurons expressing DNCDH *in vitro* show disrupted centrosome dynamics and non-muscle myosin IIB localization, while complete elimination of CDH2 reduces their migration speed and impairs polarity. *In vivo*, interneurons lacking CDH2 are less efficient in leaving the medial ganglionic eminence and reaching the cortex, as well as in invading the cortical plate (Luccardini et al., 2013, 2015). Interestingly, the effect of CDH2 on the ability of interneurons to migrate to the cortex and colonize it seems to be cell type specific, as knockout of CDH2 in Dlx5/6 expressing cells selectively reduces the numbers of calretinin and somatostatin positive interneurons, but does not alter other interneuronal types (László et al., 2020). However, the molecular mechanisms underpinning the role of CDH2 in interneuron migration remain to be elucidated.

## Discussion

The involvement of CDH2 in every step of radial migration and its function in interneurons underscore the importance of this adhesion molecule in mediating cell-cell interactions during cortical development. However, we still have a fragmented view with different observations that need to be integrated to provide a full picture of CDH2 function during neuronal migration. It is still not known if and how the different players regulating CDH2 levels are coordinated. For example, it remains to be determined whether Rap1 and Rab GTPases act in parallel pathways of if they cooperate to regulate CDH2 surface levels. The molecular signals activated upon CDH2 adhesion are also poorly understood, and the fact that mechanisms downstream of cadherins seem to be context specific means that caution should be exerted when extrapolating from different cellular systems and assumptions should be experimentally verified. It is important to keep in mind that the timepoint of intervention, dependent on age at electroporation but also on the use of different promoters, will influence results. The timing until analysis is also important because delays in polarization might mask roles in later migration phases, and the use of different dominant negative cadherin forms, sometimes at different concentrations, will also impact the phenotypes observed. These factors might explain why locomotion was considered relatively independent of CDH2 in one study (Jossin and Cooper, 2011), while being shown to be needed for this process in others (Shikanai et al., 2011; Martinez-Garay et al., 2016). Similarly, the requirement for CDH2 adhesion during MBP transition is questioned by a recent study (Kon et al., 2019), but this might reflect separate functions of CDH2 at slightly different timepoints during this complex process. Another controversy that might be explained, at least in part, by different experimental conditions is the fact that although reelin-mediated Rap1 activation seems to be required for MBP transition (Jossin and Cooper, 2011), Dab1 deficient neurons polarize correctly, enter the cortical plate and only show defects in somal translocation (Franco et al., 2011).

A final open question is the extent to which CDH2 cooperates with other adhesion molecules. Beyond its cooperation with nectins during somal translocation, no equivalent mechanism has been described for other migration phases. Connexins 43 and 26 also provide adhesion between cortical migrating neurons and radial glia fibers (Elias et al., 2007) and, interestingly, Cx43 directly downregulates CDH2 transcription during neural crest cell migration (Kotini et al., 2018). In addition, CDH2 binds astrotactin in cerebellar migration (Horn et al., 2018), raising the possibility of a similar function in the cortex. These examples highlight the potential for functional interactions between adhesion proteins and the need to expand our studies beyond individual molecules.

## Author Contributions

IM-G wrote the manuscript and created the figure and table.

## Conflict of Interest

The author declares that the research was conducted in the absence of any commercial or financial relationships that could be construed as a potential conflict of interest.
